# Leiomyoma of the Prostate Treated via Holmium Laser Enucleation: A Case Report and Literature Review

**DOI:** 10.7759/cureus.45273

**Published:** 2023-09-14

**Authors:** Masayuki Sano, Kazutaka Narimoto, Masaki Shimbo, Kazunori Hattori, Fumiyasu Endo

**Affiliations:** 1 Department of Urology, St. Luke's International Hospital, Tokyo, JPN

**Keywords:** enlarged prostate, surgical morcellation, urethral leiomyoma, holmium: yag laser, dysuria

## Abstract

Leiomyoma is a rare tumor that arises from mesenchymal cells, with few reported cases of treatment using holmium laser enucleation of the prostate. A 74-year-old man with dysuria had a mass near the bladder neck in magnetic resonance imaging; this entity was suspected to be a leiomyoma. The patient underwent holmium laser enucleation of the prostate and one lobe was removed. However, the mass was firm and morcellation was difficult to break into small pieces. Therefore, it was fragmented via trans-urethral resection and removed with a curette. The postoperative course was favorable, with a positive clinical outcome. This case highlights the efficacy of holmium laser enucleation of the prostate in the management of prostatic leiomyoma and emphasizes its importance as a viable treatment option.

## Introduction

Leiomyoma is a rare benign prostatic tumor that originates from smooth muscle cells within the prostate gland. Typically, its management includes endoscopic, surgical, and transarterial embolization therapies [1]. In a previous case, a patient was treated with holmium laser enucleation of the prostate (HoLEP) [2]. That patient underwent a prostate biopsy due to the presence of dysuria and a nodule, despite low levels of prostate-specific antigen (PSA). In our patient, magnetic resonance imaging (MRI) suggested a leiomyoma. Because the tumor location was similar to that of benign prostatic hyperplasia and a transurethral approach was feasible, HoLEP was performed directly without a prostate biopsy. HoLEP removed the tumor precisely and selectively while preserving the surrounding prostatic tissue. The procedure involves enucleating the leiomyoma from its attachment within the prostate, thereby ensuring complete excision and reducing the risk of recurrence. However, the mass was so dense that it could not be aspirated with the VersaCut™ Tissue Morcellator System (Boston Scientific, Marlborough, USA), requiring additional transurethral resection of the transected mass. In this report, we describe this case.

## Case presentation

A 74-year-old man presented to our hospital complaining of prolonged dysuria. He had pre-existing hypertension, sleep apnea syndrome, and insomnia. The PSA level was 1.92 ng/dL, the international prostate symptom score (IPSS) was 16, and his quality of life (QOL) score was 3. The patient had a maximum urine flow rate of 6.2 mL/s, an average flow rate of 4.4 mL/s, a residual urine volume of 20 mL, and a prostate volume of 53 mL.

**Figure 1:**
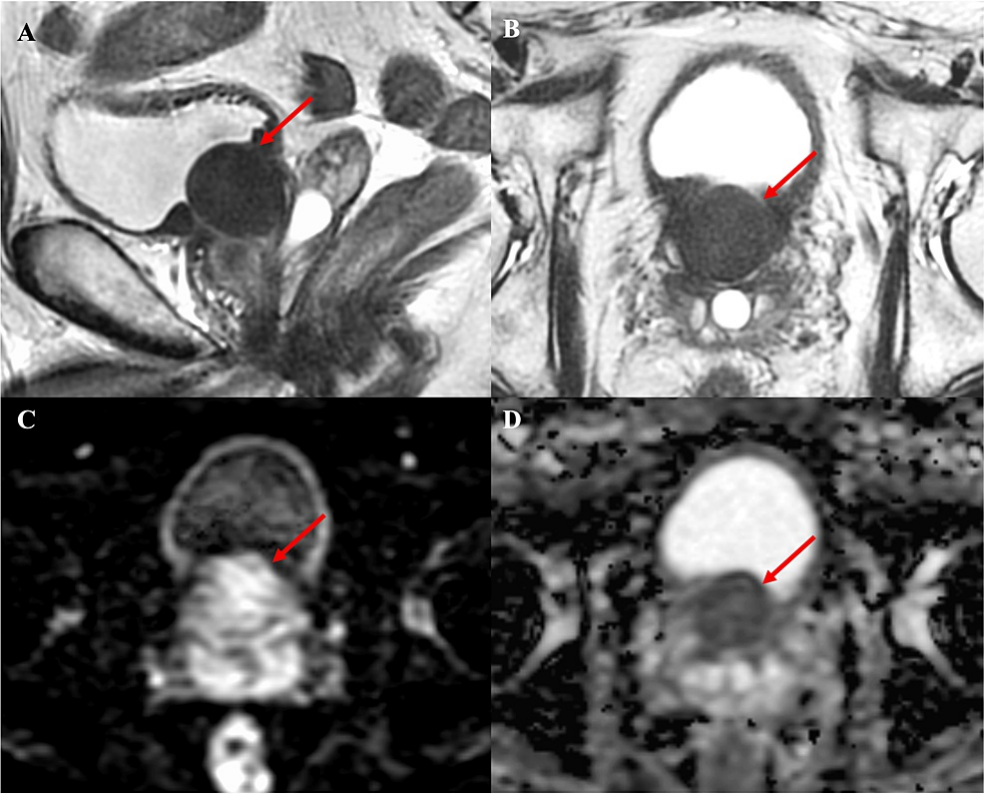
MRI of the prostate T2-weighted axial (A) and sagittal (B) images show a mass with a clear border and low signal intensity. (C, D) Diffusion weighted imaging (DWI) and apparent diffusion coefficient (ADC) mapping show diffusion restriction.

MRI revealed a 3 cm mass in the bladder neck protruding toward the bladder, with a clear border and low signal in T2-weighted images (Figure 1).

A leiomyoma of prostatic origin was suspected.

The patient subsequently underwent HoLEP and one lobe was removed during the procedure. However, the mass was very firm, which presented challenges during the morcellation process. Therefore, it was fragmented by transurethral resection and collected with a curette.

The operating time was 46 min, the bleeding was minimal, and the weight removed was 7 g. The Foley catheter was removed on the second postoperative day, and the patient was discharged on the third postoperative day.

**Figure 2:**
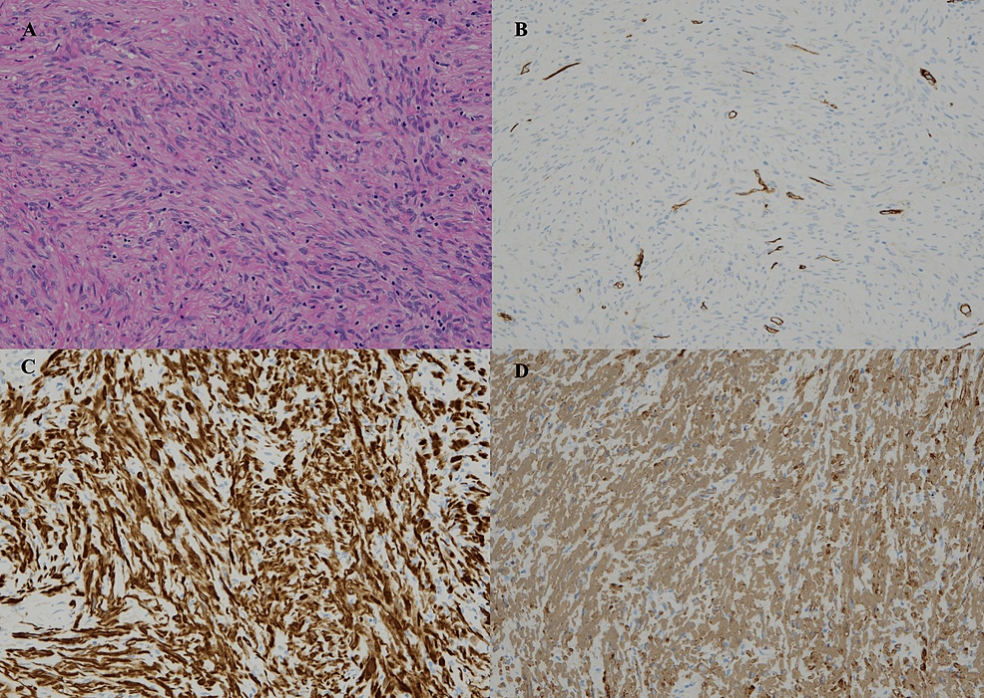
Microscopic studies (A) spindle cell proliferation with no necrosis and no prominent mitotic pattern (H.E. ×10). (B) CD34 staining showing no reactivity. (C) Desmin staining is positive. (D) Strong αSMA staining is seen.

Pathologically, the enucleated prostate mass consisted of proliferated spindle cells with no epithelial involvement; the cytoplasm was eosinophilic, and there was no characteristic arrangement. There was no necrosis and the mitotic pattern was unremarkable (0/10 high power fields) (Figure 2).

Immunostaining indicated that the tissue was Desmin+, αSMA+, CD34-, STAT6-, CD117-, S100-, and PSA-. These findings were suggestive of a leiomyoma.

Three months postoperatively, the patient’s maximum urine flow rate had increased to 16.1 mL/s and the volume of residual urine had decreased to 4 mL. The IPSS improved markedly to 4, while the QOL score improved to 1. There were no significant postoperative complications. After one year following the surgery, there has been no sign of recurrence.

## Discussion

Leiomyomas originating in the prostate are rare, and very few cases have been treated endoscopically, especially via HoLEP [2]. We experienced a patient who presented with severe dysuria and successfully underwent endoscopic surgery to achieve excellent urinary voiding.

Histologically, leiomyomas have well-organized fascicles, which are rarely observed in stromal nodules. Furthermore, they have minimal to no nuclear atypia, virtually no mitotic activity, and occasionally have scattered degenerative nuclei within a normocellular background [3]. Immunohistochemically, leiomyomas are strongly positive for myogenic markers such as desmin and actin [4]. Furthermore, unlike stromal tumors of unknown malignant potential, CD34 is not expressed [1]. Our case was consistent with these findings.

Advances in preoperative imaging studies have made it possible to diagnose leiomyoma with greater precision [5]. MRI shows the distinct features of leiomyoma, including a well-defined pelvic mass originating from the prostate that lacks invasive characteristics. Ultrasound is nonspecific and shows a mass that can be hyper- or hypoechoic. The masses in computed tomography (CT) and MRI can either be homogenous or contain areas of cystic degeneration. Non-cystic areas appear isodense to muscle in CT, while in MRI they are T1-isointense and T2 hypo to slightly hyperintense relative to muscle. Diffusion weighted imaging (DWI) reveals restricted diffusion with a low apparent diffusion coefficient (ADC) value [6]. The MRI features and PSA values enable a diagnosis of leiomyoma. In our case, the diagnosis of leiomyoma was confirmed preoperatively based on the MRI findings and low PSA, which made it possible to perform HoLEP directly without a biopsy. However, since malignancy cannot always be ruled out based on imaging alone, preoperative needle biopsy should be considered if malignancy is strongly suspected due to findings such as irregular morphology or high PSA.

The use of HoLEP in the treatment of prostatic leiomyoma offers several advantages over traditional surgical methods. It allows enhanced visualization and accessibility, enabling the precise removal of the tumor, while minimizing complications such as bleeding and damage to surrounding structures that causes urinary incontinence. [7]

Moreover, HoLEP offers a shorter hospital stay and faster recovery time compared to open surgery, making it an appealing choice for patients, and can be used to treat leiomyomas in close proximity to the urethra.

In our case, the leiomyoma was well-circumscribed and could be completely removed by enucleation. However, because the tissue was very hard, morcellation using the VersaCut™ Tissue Morcellator System was difficult, and endoscopic resection was necessary to cut it into smaller pieces. To deal with this issue, an oscillation-type morcellator, which is better for extraction than a reciprocating system, may be the best choice. [8] This should be kept in mind when encountering such cases in the future. In this case, the procedure demonstrated excellent efficacy in relieving lower urinary tract symptoms and improving urinary flow rates. Successful treatment of this benign condition eliminates the possibility of recurrence.

## Conclusions

In conclusion, prostatic leiomyoma associated with dysuria can be managed effectively with the use of HoLEP. Accurate preoperative diagnosis by imaging is possible and helps determine the surgical approach. However, the histological hardness of leiomyoma must be taken into consideration, and appropriate morcellation machines must be utilized. Consequently, HoLEP is a valuable therapeutic modality for prostatic leiomyoma due to its demonstrated efficacy, safety, and positive patient outcomes.
